# Minimal Open Access Ileocolic Resection in Complicated Crohn's Disease of the Terminal Ileum

**DOI:** 10.1155/2020/6019435

**Published:** 2020-02-28

**Authors:** Giuseppe S. Sica, Sara Di Carlo, Stefano D'Ugo, Claudio Arcudi, Leandro Siragusa, Laura Fazzolari, Livia Biancone, Giovanni Monteleone, Maurizio Cardi, Simone Sibio

**Affiliations:** ^1^Department of Surgery, Tor Vergata University of Rome, Tor Vergata Hospital-Viale Oxford 81, 00133 Rome, Italy; ^2^Department of Medicine, Tor Vergata University of Rome, Tor Vergata Hospital, Viale Oxford 81, 00133 Rome, Italy; ^3^Department of Surgery “Pietro Valdoni”, Sapienza University of Rome, Umberto I Hospital-Via Lancisi 2, 00155 Rome, Italy

## Abstract

The objective of this study was to evaluate the possibility to undertake an ileocolic resection in complex Crohn's disease using a minimal open abdominal access using standard laparoscopic instruments. The incision was carried out over the previous McBurney scar, with a mean length of 6 cm. Seventy-two patients with complicated Crohn's disease underwent IC resection in the considered period; 12 patients had a McBurney scar due to a previous appendectomy and represented the group of study. Feasibility and safety of the procedure were evaluated. Clinical data and outcome were compared with a control arm of 15 patients who had a standard laparoscopic IC resection, pooled out from our database among those who had a McBurney incision as service incision. Mean operative time and postoperative stay were significantly shorter in the study group. Blood loss and operative costs were also lower in the study group but did not reach statistical significance. Minimal open access ileocolic resection (MOAIR) through a small McBurney incision seems safe and feasible in complex Crohn's disease. Some advantages over standard laparoscopic surgery could be found in surgical outcomes and costs.

## 1. Introduction

Laparoscopy is the preferred surgical approach for ileocolic (IC) resection in Crohn's disease (CD) when appropriate expertise is available [1, 2]. It was first reported by Milson et al. in 1993 [3], and since then gained popularity and acceptance even for complex cases [1, 4]. Despite the increasing evidence of its safety and efficacy, concerns have been raised about its widespread feasibility especially in patients with previous abdominal surgery and in more complex cases (perforating or recurrent CD), where high conversion rates are still reported even in high volume referral centers [5].

In patients with previous abdominal surgery, it is often convenient to use the existing scar as the preferred service incision. In particular, in patients who had previously an open appendectomy, the McBurney scar represents an ideal abdominal site to be used as service incision and, in most instances, the very first access to the abdominal cavity if a device such as the Alexis O wound protector/retractor (Applied Medical, 22872 Avenida Empresa, Rancho Santa Margarita, CA92688 USA) is to be used.

The aim of this study was to evaluate feasibility and safety of small McBurney incision for ileocolic resection in CD patients who already undergone appendectomy through a previous McBurney incision. MOAIR is compared with our standard laparoscopic approach in a similar group of patients to find differences in the outcome.

## 2. Materials and Methods

### 2.1. Patients

From January 2016 to December 2017, we prospectively considered all consecutive patients with primary or recurrent CD referred to our Minimally Invasive and Gastrointestinal Surgery Unit for IC resection. The disease was defined complex in the presence of fistulas and abdominal abscess; a very thickened mesentery or large inflammatory masses determining adhesions were also reported. The study group was composed of patients with complicated CD presenting with an existing McBurney incision for a previous open appendectomy undergoing MOAIR. The control group consisted of CD patients with comparable clinical characteristics who underwent a standard laparoscopic IC resection using a service incision the same pre-existing McBurney. Clinical data were collected for the study purpose and reported in Table 1. The characteristics of the population of study were divided into demographics, such as age and gender, and clinical data, such as body mass index (BMI), type and behavior of CD, duration of disease, presence of extra ileocecal disease, and previous surgery, past, and current medical treatment. Data of the perioperative period, including preoperative imaging results (magnetic resonance enterography or small intestine contrast ultrasound) and concordance with the intraoperative findings were recorded. Intraoperative data consisted of operative time, blood loss, conversion rates (open to laparoscopy or laparoscopy to open surgery), complications and complexity of the disease according to the presence of abscesses and fistulas, and/or large masses and thickened mesentery. Finally, patients were operated under our protocol of enhanced recovery in abdominal surgery (ERAS) which follows the basic principle of the ERAS [6], and adherence to the protocol was evaluated in the two groups. Recurrence at one year has been reported. Differences between groups were statistically analyzed by mean of the univariate chi-squared test analysis tool (NCSS (Number Cruncher Statistical Software), 329 North 1000 East Kaysville, Utah 84037, USA). A *p* value of <0.05 has been set as significant. Written informed consent was obtained from all patients.

### 2.2. Surgical Technique

#### 2.2.1. Study Group

**Figure 1:**
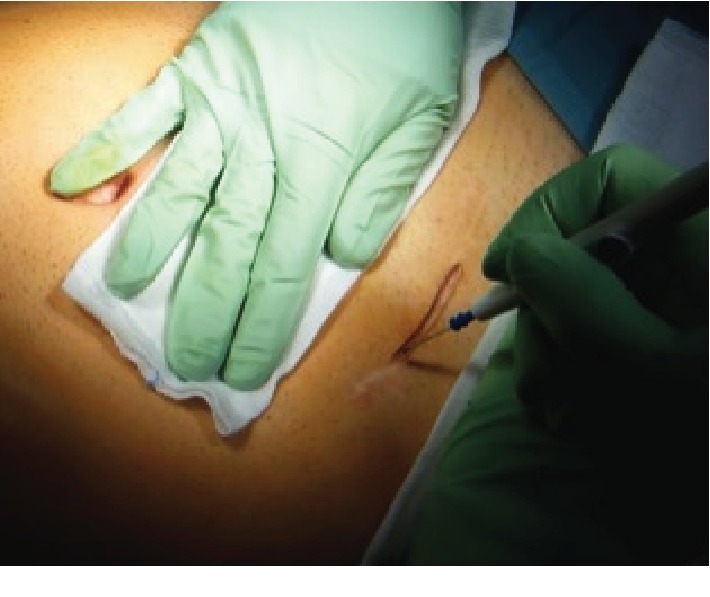
Skin incision on the previous McBurney.

**Figure 2:**
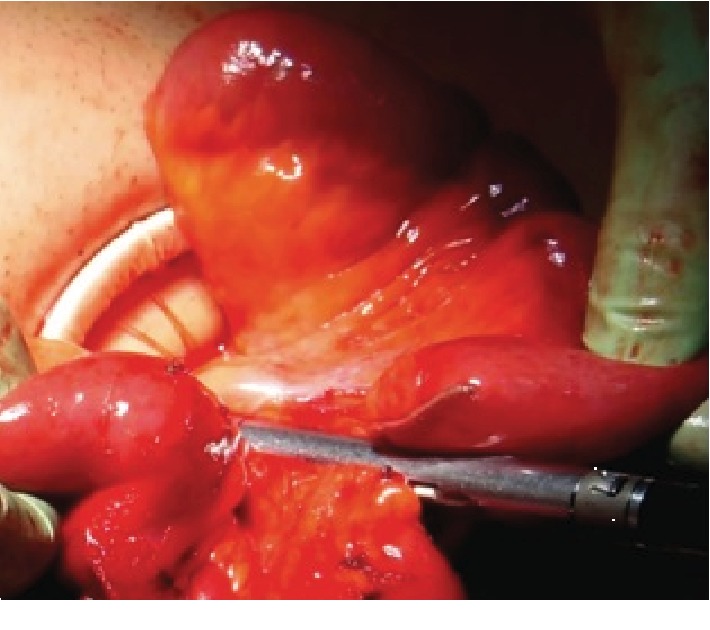
Resection of the ileum and mesentery.

**Figure 3:**
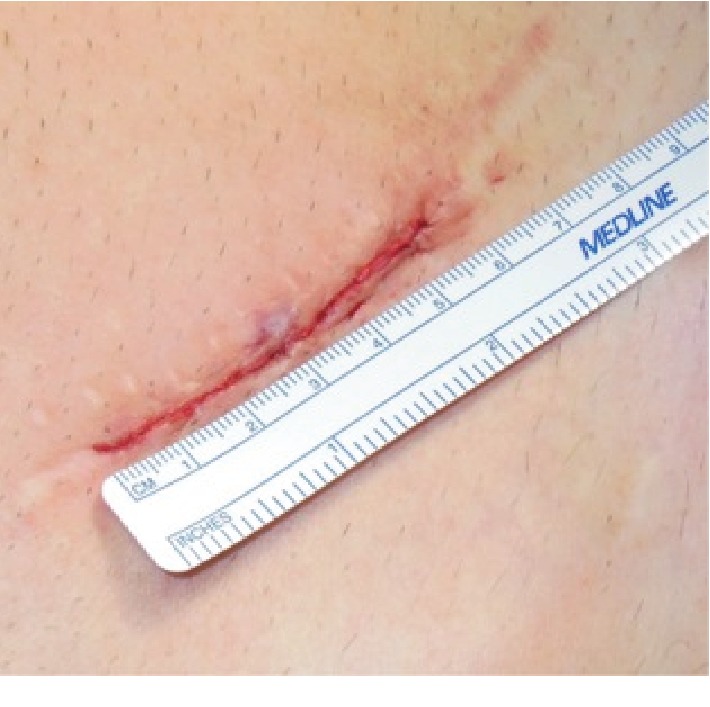
Wound closure.

The operating room was set as per standard laparoscopic IC resection with the laparoscopy tower available and the scrub nurse table ready for a quick “conversion.” Patients were intubated and subsequently placed in a supine position with the legs opened, the surgeon standing at the patient's right side or between the legs. Skin incision was done over the McBurney scar (Figure 1). Once access to the abdominal cavity was gained, the small size (2.5-6 cm) Alexis was used to protect and retract the wound margins and to allow laparoscopy. The surgical procedure followed the same principles of benign colorectal surgery. The surgeon normally standing at the patient's right side is ready to move between the legs if necessary especially during the upper part of the colonic dissection. A careful inspection of the ileum using Rampley forceps was performed. In case of adhesions, a mix of sharp and blunt dissection was carried out through the incision, also with the aid of a laparoscopic ultrasound harmonic scalpel (Ultracision™, Ethicon Endosurgery). In case of true penetrating fistulas into the bladder or colon, the sutures were carried out from the incision. Once freed, the terminal ileum was divided using an endoscopic stapler (Figure 2). The cecum and ascending colon were mobilized and the hepatic flexure lowered (if not already done in the previous operation), dividing the retroperitoneal attachments. The ileocolic vessels or their branches were divided using the harmonic scalpel. The hepatic flexure was mobilized only if necessary and the specimen (ileocolic complex) exteriorized through the ring; part of the greater omentum was generally divided while performing the division of the gastrocolic attachments. The ascending or transverse colon was then stapled to complete the ileocolic resection. Finally, a side-to-side antiperistaltic mechanic ileocolic anastomosis was fashioned and the new ileocolic complex was placed back into the abdomen. According to our ERAS protocols, the stomach was never drained and the abdominal drains were placed only in the presence of abscesses or fistulas. A Foley catheter was inserted only if required by the anesthesiologist. A subcuticular suture or staples were used to close the skin (Figure 3). Intraoperative fluid restrictions and sparse use of opioids were applied whenever possible. Patients were allowed to drink fluids and eat biscuits up to 4 hours before surgery and in the evening after the operation or eventually the following morning.

#### 2.2.2. Control Group

In the laparoscopic group, the technique is slightly different from our standard laparoscopic IC resection, as usually the first access would be through a periumbilical incision, and the Alexis O wound protector/retractor trocar was used to insufflate and maintain the pneumoperitoneum. In these patients, the McBurney incision is used as the first access and the Alexis trocar is used to obtain the pneumoperitoneum. Two ports, one for the camera and one 5 mm working port, are placed in the left flank. Dissection of the cecum and ascending colon, hemostasis, and resection of the ileum are done laparoscopically, the resection of the colon and anastomosis through the service incision. No laparoscopic control is routinely performed at the end of the procedure.

## 3. Results

### 3.1. Demographics and Clinical Data

Demographics and clinical data of the two groups are reported in Table 1. In the two years of the study period, 72 patients with complicated ileocecal CD were referred to our institution for surgical treatment. Twelve have already undergone appendectomy through a McBurney incision, underwent MOAIR, and were considered for the study purposes. The control group was made of 15 patients with similar demographic and clinical characteristics who underwent a standard laparoscopic IC resection with the service access through the McBurney incision. Groups were similar for age, sex, and BMI. Mean duration of CD (time since first diagnosis) was 37.6 months (range 8-96) in the first group and 41.2 months (range 9-102) in the control group (*p*: n.s.).

### 3.2. Surgical Outcomes

Mean operative time was 89 minutes (range 75-156) in the study group and 122 minutes (range 91-148) in the control arm (*p*: 0.03). Mean blood loss was 128 mL (20-450 mL) in the study group and 137 mL (range 25-512) in the control group (*p*: 0.051). All patients in both groups were compliant to our ERAS protocol. Mean postoperative hospital stay was significantly lower in the study group (4 days, range 3-7) when compared with that in the control group (6 days, range 4-9) (*p* < 0.05). No delay in LOS was observed in patients carrying abdominal drains.

### 3.3. Study Group

In the study group, two patients had already undergone a previous laparoscopic ileocolic resection. The first patient had an anastomosis stricture and a mesenteric abscess; the second had a large pelvic inflammatory mass involving the neoterminal ileum, the iliac muscle, and both the posterior and anterior abdominal walls. In both patients, the right colonic flexure was already lowered. The other 10 patients were naïve for surgery: 4 had penetrating disease (into sigmoid colon, mesocolon, and small bowel) in three cases with small mesenteric abscesses. The mesentery was extremely thickened in 4 cases, and in 2, the prestricture small bowel was dilated and thickened. The mean length of the surgical incision was just less than 6 cm, often shorter than the existing McBurney scar. Good correspondence was found between preoperative imaging and intraoperative findings. Five patients had a drain left at the end of the surgery: four had a mesenteric abscess and one a very large inflammatory mass; the drain was removed in the first postoperative day in three cases and the second postoperative day in two. Clinical characteristics were similar in the control arm, with 3 patients with recurrent disease. In the study group, it was possible to complete the MOAIR in all 12 patients without any conversion to laparoscopy.

### 3.4. Control Group

In the control arm, there were 2 late conversions (more than fifteen minutes after trocars insertion) to open surgery: the first patient had a difficult mobilization of a large inflammatory mass stuck to the posterior abdominal wall and pelvis; and the second patient had uncertain anatomical landmark due to previous surgery and recurrent fistulising disease. In four patients, the abdomen was drained after the procedure and the drains were removed within 36 hours. No intraoperative complications were observed in this series.

### 3.5. Morbidity and Mortality

Regarding postoperative complications in the study group, only one patient had a minor (Clavien-Dindo II) adverse event, a wound infection treated with antibiotics. In the control group, we observed three minor complications, two wound infections and one urinary infection treated with antibiotics (*p*: n.s.). No mortality was observed in both groups.

### 3.6. Follow-Up

One patient in the study group and one in the control group had an endoscopic and mild clinical recurrence at the 1-year follow-up.

## 4. Discussion

Laparoscopy should be the preferred approach for ileocolic resection in CD when appropriate expertise is available [1]. We already reported our experience with laparoscopic ileocecal resection in the nonselected patients with complicated CD and disease behavior, complexity of cases, conversion rates, perioperative complications and patient's satisfaction-confirmed feasibility, and advantages of the minimally invasive approach [7]. Three meta-analyses including 15 studies confirmed the consistent benefit of laparoscopy [1, 4, 8]. Moreover, patients with CD laparoscopic resections share the same long-term recurrence rate when compared with those with the open approach, eliminating the concern of missing occult segments during laparoscopy [9–12].

However, in case of complex cases or for recurrent disease, the recommendations in favor of the laparoscopic surgery are not so strong [2, 4, 13]. In a recent meta-analysis reporting seven nonrandomized studies, laparoscopic surgery for recurrent CD is recommended only in selected cases, and conversion rate is significantly higher when compared with naïve CD patients [14].

Our study describes the use of a novel minimally invasive open technique for ileocolic resection in a group of patients with complex CD who previously underwent an open appendectomy through a McBurney incision. The underlying idea is that an open but minimal surgical access could provide advantages such us avoiding “late” conversions and reduction in operative time and costs, together with the known advantages of minimal invasive surgery. Clinical outcome was compared with a control group of patients with similar clinical characteristics but treated with a conventional laparoscopic approach. The results seem to suggest that a small right inguinal access, conveniently made over the McBurney scar, is *good enough* to undertake a complex ileocolic resection for CD. Using a pre-existing incision as the only abdominal access, this technique may also offer cosmetic advantages over conventional multiport laparoscopic surgery. The choice of an open surgery through a McBurney incision is compliant with the ECCO guidelines that report insufficient evidence to recommend laparoscopic surgery as the first choice in more complex cases [1, 4].

The standard laparoscopic approach usually requires three to four ports, with the related risks of visceral and vascular injuries, port-site complications (bleeding, postoperative abdominal wall hematomas, local nerve irritation, and port-site incisional hernia), and postoperative pain. A service laparotomy is usually necessary in almost all techniques, and in case of an existing abdominal incision, it is usually the chosen service access to the abdomen. In our experience, we noted that in the patients with a previous open appendectomy, the McBurney incision used to perform a single-port laparoscopic ileocecal resection was good enough to dissect and mobilize both the small bowel and the right colon [15]. In the first five cases of this series, an exploratory laparoscopy was carried out to inspect the abdominal cavity at the end of the procedure. From case five, even though the laparoscopic equipment was ready in the operating room, no laparoscopy was performed after the resection, as it is for almost all our laparoscopic ileocecal resections. The incision that was made over the McBurney scar was almost always shorter than the existing scar even in overweight patients. The Alexis O wound protector was useful to access the abdominal cavity and made it possible to easily convert to laparoscopy. The McBurney incision gives a good exposure to the operative field and offer a safe visual of all the important structures, such us the ileocolic vascular pedicle, right ureter, and duodenum.

Although laparoscopy is currently considered safe and feasible in the primary Crohn disease and experienced authors recommend its application even in more complex cases [16], high rates of conversion are still reported (up 25.4%) [5, 16]. In this small series, a McBurney incision, together with the extensive use of Alexis O wound retractor, provided optimal exposure of the surgical field also in cases of penetrating the disease; in the study sample, we have operated four patients with fistulas to sigmoid colon, ileum, and mesocolon. However, it is not possible from such a small incision to explore the whole abdomen as it is in laparoscopy, but certainly, there is no constriction regarding site view and possibly a fistula between the left side of the bladder or the sigmoid colon can be handled even easily this way. For what concerns the potential risk of missing lesions, we found an almost 100% matching between the results of preoperative imaging (EUS, CT, and MRI) and intraoperative findings, and this is also the reason why we do not routinely explore the abdominal cavity during our laparoscopic ileocecal resections.

The MOAIR seemed a straightforward operation, easy to perform even in complex cases. As stated, it was initially thought as part of a laparoscopic ileocolic resection, after the insertion of the Alexis ring port over the McBurney incision. Eventually, it was clear that, in most instances, the sole incision was enough to undertake the whole operation. From this observation, a comparative analysis was planned. Our results showed that MOAIR was feasible and safe in this preliminary series, demonstrating some advantages such as shorter mean operative time and mean hospital stay, while no significant differences were noted in blood loss, conversion rates, and postoperative complications. In the control group, a conversion to open procedure was necessary in two patients due to large inflammatory mass and pelvic abscesses. The conversion rate (13%) is in line with current literature reports, but higher than the 2% reported by Nguyen et al. [17]. In their recent paper, Mege and Michelassi described their experience on 427 consecutive patients undergoing abdominal surgery for CD and concluded that increasing expertise in laparoscopic surgery carry a decrease in the conversion rate, even in more complex cases. Predictive factors for conversion included older age, current smoker, recurrent disease, thickened mesentery, and large inflammatory mass. Despite the vast experience with both CD and laparoscopic surgery, they concluded that one-fifth of the cases still need conversion [5]. It is possible that different results among authors are due to different patient selections. In fact, in this series, only patients with complex CD (recurrent, fistulizing, multiple fistulas, thickened mesentery, and pelvic abscess) and not those with simple stenosis of the terminal ileum are included, as reported by other authors [18]. This study may add some additional evidence in the preoperative decision of whether a patient with complex CD should be approached through a conventional laparoscopic or open access.

Patients in both groups received the same perioperative care, and in both the study and the laparoscopic group, patients were compliant to the proposed ERAS protocol. As shown by many studies, following the principles of enhanced recovery conveys advantages in the postoperative recovery with a shorter hospital stay and lower overall complication rates [19]. Even if according to ERAS recommendations, the placement of a drain should be avoided, in patients with abdominal abscesses or large masses, we preferred to leave in place a drain for one or two days. The perceived need for the placement of an abdominal drain was not different in the two groups (five patients in the study group and four in the control group) and did not affect postoperative stay.

## 5. Conclusions

MOAIR for complex CD seems to be safe and feasible in patients with an existing McBurney incision and, in our small series, it seems to reduce operative time and length of hospital stay, with similar complication and conversion rates when compared with a standard laparoscopic approach.

## Figures and Tables

**Table 1:** Demographics and surgical outcome factors of the two samples (univariate analysis–chi-squared test).

Factor	Study (*n* = 12)	Control (*n* = 15)	*p*
Mean age (years) (range)	40 (25-60)	38 (22-61)	0.08
Gender (%)			
Male	8 (67%)	9 (60%)	0.09
Female	4 (33%)	6 (40%)	
Mean BMI (kg/m^2^)	23 (18-27)	24 (19-28)	0.1
Behavior of disease (%)			
Primary	10 (83.3%)	12 (80%)	0.2
Recurrent	2 (16.7%)	3 (20%)	0.1
Fistulas+pelvic sepsis	6 (50%)	9 (60%)	0.07
Thickened mesentery	4 (33%)	4 (27%)	0.06
Large inflammatory mass	2 (17%)	2 (13%)	0.1
Mean length of disease (months) (range)	37.6 (8-96)	41.2 (9-102)	0.07
Medical treatment	7 (58%)	8 (53%)	0.09
Steroids	3 (43%)	5 (62%)	0.06
Aminosalicylate	4 (57%)	3 (38%)	0.07
Mean op. time (min)	89 (75-156)	122 (91-148)	**0.03**
Mean blood loss (mL)	128 (20-450)	137 (25-512)	0.051
Conversion			
LPS	—		
Open		2 (13%)	
Mean hospital stay (days)	4 (3-6)	6 (4-9)	0.04
Morbidity (CD) (%)			
I	—	—	
II	1 (8%)	3 (20%)	0.06
III	—	—	
IV	—	—	
Adherence to ERAS (%)	10 (83%)	13 (87%)	0.1
1-year recurrence (%)	1 (8%)	1 (7%)	0.06

LPS = laparoscopy; BMI = body mass index.

## Data Availability

The data used to support the findings of this study are included within the article.
